# Can single cell RNA sequencing reshape the clinical biochemistry of hematology: New clusters of circulating blood cells

**DOI:** 10.1002/ctm2.671

**Published:** 2021-12-12

**Authors:** Hao Fang, Yiming Zeng, Lianzhong Zhang, Chengshui Chen, Charles A Powell, Xiangdong Wang

**Affiliations:** ^1^ Department of Anesthesiology Zhongshan Hospital Fudan University Shanghai China; ^2^ Department of Anesthesiology Minhang Hospital Fudan University Shanghai China; ^3^ Department of Pulmonary and Critical Care Medicine Clinical Center for Molecular Diagnosis and Therapy Second Affiliated Hospital of Fujian Medical University Respiratory Medicine Center of Fujian Province Quanzhou China; ^4^ Henan Provincial People's Hospital People's Hospital of Henan University People's Hospital of Zhengzhou University Zhengzhou China; ^5^ The Quzhou Affiliated Hospital of Wenzhou Medical University Quzhou People's Hospital Quzhou Zhejiang China; ^6^ Division of Pulmonary Critical Care and Sleep Medicine Icahn School of Medicine at Mount Sinai New York New York USA; ^7^ Department of Pulmonary and Critical Care Medicine Shanghai Institute of Clinical Bioinformatics Shanghai Engineering Research for AI Technology for Cardiopulmonary Diseases Jinshan Hospital Centre for Tumor Diagnosis and Therapy Fudan University Shanghai Medical College Shanghai China

## Abstract

scRNA‐seq is on track for use as a routine measurement of clinical biochemistry and to assist in clinical decision‐making and guide the performance of molecular medicine, but there are still a large number of challenges to be overcome. In conclusion, scRNA‐seq‐based clusters and differentiation of circulating blood cells have been examined and informative in patients with various diseases, although the information generated from scRNA‐seq varies between different conditions, technologies, and diseases. Most of the clinical studies published have focused on the landscape of circulating immune cells, disease‐specific patterns of new clusters, understanding of potential mechanisms, and potential correlation between cell clusters, differentiations, cell interactions, and circulating and migrated cells. It is clear that the information from scRNA‐seq advances the understanding of the disease, identifies disease‐specific target panels, and suggests new therapeutic strategies. The adaptation of scRNA‐seq as a routine clinical measurement will require standardization and normalization of scRNA‐seq‐based comprehensive information and validation in a large population of healthy and diseased patients. The integration of public databases on human circulating cell clusters and differentiations with an application of artificial intelligence and computational science will accelerate the application of scRNA‐seq for clinical practice. Thus, we call special attention from scientists and clinicians to the clinical and translational discovery, validation, and medicine opportunities of scRNA‐seq development.

Can single‐cell RNA sequencing (scRNA‐seq) act as a clinical biochemical routine measurement approach for diagnosing and evaluating the severity, stage, and specificity of disease as well as patients’ response to therapy? Can the comprehensive information obtained from scRNA‐seq assist in clinical decision‐making for therapeutic strategies and guide the performance of precision medicine? Circulating blood cells are an important tissue component essential to maintaining systemic hemostasis, interorgan communications, and responses to pathogens, and analysis has been applied in critical and routine hematological measures in clinical biochemistry. The number, composition, and differentiation of circulating blood cells represent important components of clinical evaluation. The measurement techniques of circulating blood cells mainly include cell morphology, chemical staining, and immunochemical labeling. The clusters of the differentiation (CD) nomenclature system were established based on monoclonal antibody classification of antigens on the surface of circulating blood cells, using the consensus made by the First International Workshop on Human Leucocyte Differentiation Antigens in 1982.^1^ Clusters of circulating leukocytes are identified and classified using monoclonal antibodies and presented as the number and percentage of leukocytes by positive staining of immunofluorescence.

With the rapid development of biotechnology, scRNA‐seq has emerged as a breakthrough approach to the identification and validation of disease biomarkers and as a critical analytic approach in single‐cell biology. The methodology of scRNA‐seq is still under development and is quickly improving in response to clinical requests and practice, including approaches important for sampling, restoration, quality control, sequencing depth, data mining and analysis, clinical interpretation, and cost. In single‐cell biomedicine, DNA sequencing in single‐cell nuclei and single‐cell RNA sequencing together can present comprehensive profiles of cellular membrane, intracellular organelles, cytoplasmic factors, and nuclear elements in physiological and pathological conditions.^2^ The number of human studies on scRNA‐seq of circulating blood cells in numerous diseases is growing quickly. Certainly, scRNA‐seq has great value in the identification of disease‐specific biomarkers, which after preclinical and clinical validations can be developed as accessible diagnostic kits. From the point of view of clinical and translational medicine, questions to consider are: 1. whether the method of scRNA‐seq is repeatable, stable, and reliable enough for clinical application; 2. what value is added from scRNA‐seq data to assist clinical decision making; 3. whether the characterization of circulating blood cells generated from scRNA‐seq can augment the CD nomenclature system; and 4. how CDs produced from scRNA‐seq can be validated and standardized as a clinical biochemical analytics package for routine hematological analysis.

New clusters of circulating blood cells have been identified by both scRNA‐seq alone and in combination with other methodologies, for example, mass cytometry, bulk RNA sequencing, flow cytometry, or proteomics. The combinations provide comprehensive profiles on the cell clusters with higher dimensional characterization and correlation with biological functions, and new insights for understanding the significance of new clusters. Although these approaches are unlikely to be performed in routine clinical practice, they will be important for optimizing and standardizing the clinical importance of scRNA‐seq‐based data in the context of disease severity, duration, and response to therapy.

scRNA‐seq is emerging as an important tool to understand systemic immune responses to emergent diseases such as COVID‐19 by identifying new clusters of circulating cell differentiation, and by elucidating mechanisms of disease pathogenesis. In a single‐cell atlas of circulating blood cells harvested from patients with COVID‐19, scRNA‐seq‐based discoveries of transcriptomic profiles of peripheral blood mononuclear cells (PBMCs) from patients with COVID‐19 were measured to understand molecular pathways in peripheral immune cells and define new clusters and differentiations in patients with COVID‐19. Wike et al demonstrated the depletion of γδ T cells, plasmacytoid dendritic cells (pDCs), and CD56^bright^ natural killer (NK) cells, as well as the increase of plasmablasts in 7 patients with severe COVID‐19, as compared with those with typical acute respiratory distress syndrome or healthy controls.^3^ Schulte‐Schrepping et al measured single‐cell transcriptomic and proteomic profiles of 242 whole‐blood and PMBCs from 109 patients with mild or severe COVID‐19 and found emergency myelopoiesis, dysfunctional mature neutrophils, and HLA‐DRlo monocytes as biomarkers for severe COVID‐19.^4^ In this particular study, distinct subsets of CD14^+^HLA‐DRAhi, CD14^+^HLA‐DRB1^hi,^ and CD14^+^CD83^hi^ monocytes were detected in mid‐COVID‐19, while CD24^+^, PGLYRP1^+^, DEFA3^+^, DEFA4^+^, FCGR3B^+^, CXCL8^+^, and LCN2^+^ neutrophils were detected in severe COVID‐19. The multi‐transcriptomic sequencing of noncoding RNAs and mRNAs from patients with moderate and severe COVID‐19 demonstrated that the noncoding and coding transcriptional landscape of peripheral immune cells provided novel insights into COVID‐19 pathogenesis.^5^

With the combination of bulk transcriptome and DNA methylome analysis, scRNA‐seq was applied to the analysis of more than 358,000 peripheral blood cells dynamically at days 0, 2, 7, 10, 13, and/or at discharge, during which clusters of monocytes, proliferative lymphocytes, and NK cells were altered.^6^ Of these clusters, CD14^+^ monocytes increased during convalescent stages, nonclassical CD16^+^ monocytes, plasmablast proportions during incremental and early convalescence, and several bone marrow‐derived precursor cell types increased in patients with COVID‐19. scRNA‐seq‐based new clusters of each circulating blood cell differentiation were investigated in patients with COVID‐19, including whole blood cells, PBMCs, monocytes, and lymphocytes. It indicates that scRNA‐seq becomes more and more important in identifying new clusters of circulating blood cells, cluster‐specific networks, function‐based mechanisms, and measurable targets of critical illnesses, serious infectious diseases, and major pandemics. Integration of single‐cell TCR sequencing, deep TCR repertoire sequencing, and HLA genotyping, scRNA‐seq of PBMCs from early‐recovery COVID‐19 patients with demonstrated novel single‐cell gene expression, paired αβTCRs, and hypervariable regions of immune receptors, that identified potential novel vaccine targets for COVID‐19.^7^ Major challenges include difficulties performing well‐designed studies with sufficient sample size, controlling confounding by severity, duration, stage, and genetic background of diseases, and defining clear correlation and interaction between clusters and differentiation. Nevertheless, there is great potential for developing disease‐specific panels of circulating blood cell clusters and differentiations to predict risk or to screen for diagnosis in human populations.

With rapidly increasing CD numbers, the clinical laboratory has more potentially measurable cell surface antigens and discrete cell differentiation states, although the number of labeled cell differentiations and clusters per time are still limited and a large amount of blood volumes are needed for an increased number of cell differentiations, frequencies, and phenotypes. It is a challenge to have so many immunophenotyping reagents available for flow cytometry, simultaneously measuring multiple cell differentiations, and uncovering unknown clusters of differentiation. scRNA‐Seq can break through those barriers by characterizing circulating blood cell clusters, differentiation, molecular function, and biological interactions. Up to 100 circulating blood cell clusters and differentiation can be comprehensively identified using scRNA‐seq, and these can vary among species, ages, sexes, diseases, severities, and stages, and especially between acute and chronic disease. Song et al measured intracellular and cell surface proteomic profiles and circulating immune cell bulk RNA and single‐cell transcriptomic profiles of patients with stable pneumonia, unstable pneumonia, stable asthma, acute asthma, acute exacerbation of the chronic obstructive pulmonary disease, chronic obstructive pulmonary disease, and lung cancer, by combining scRNA‐seq with flow cytometry, mass cytometry, and bulk RNA‐seq.^8^ This study showed that altered patterns of clusters and differentiations of circulating immune cells differed between diseases, between acute and chronic stages, between allergy and infection, and between chronic pathology and cancer, although the specificity of scRNA‐seq‐based clusters and differentiations remains to be further validated in larger independent populations of patients.

In cancer, scRNA‐seq has been widely applied to identifying new clusters of inflammatory cells in human tissue and intercellular interactions within the microenvironment. In addition to better understanding molecular mechanisms of how inflammatory cells contribute to tumorigenesis and progression of cancer, scRNA‐seq has been applied to understand if t pathology‐specific clusters of tissue inflammatory cells have definite and dynamic associations with circulating leukocytes that are mirrored by CDs of circulating cells. scRNA‐seq was applied to mapping the landscape of tumor cells and inflammatory cells within the tumor before and after therapy and at different stages of cancer.^9^, ^10^ Clusters and differentiations of tissue inflammatory cells varied among disease natures, stages, dynamics, and responses to therapy.

Other studies have examined whether new clusters of inflammatory cells can be measured in circulating blood cells and whether they can be mirrored by alterations of circulating blood cells with those phenotypes. For example, 18 unique cell populations of inflammatory cells (e.g., T cells, B cells, monocytes) were identified in synovial tissue from patients with rheumatoid arthritis or osteoarthritis based on single‐cell transcriptomic profiles, including interleukin 1B^+^ pro‐inflammatory monocytes, integrin alpha X^+^ T‐Box Transcription Factor Protein 21^+^ autoimmune‐associated B cells, programmed cell death 1^+^ peripheral helper T cells and follicular helper T cells, and distinct subsets of CD8^+^ T cells characterized by granzyme K^+^, granzyme B^+^, and granulysin^+^ phenotypes.^11^ Those specific cell subsets were identified using high‐dimensional multi‐modal single‐cell data from synovial tissue samples and they were found to contribute to pathways relevant to rheumatoid arthritis and chronic inflammation.

scRNA‐seq‐based phenomes of circulating immune cells were characterized in a population with chronic exposure to cadmium, where CD14^+^ monocyte clusters were reduced in proportion to the severity of exposure.^12^ Clusters and functional status of circulating CD8^+^ T cells are dependent factors for systemic immune response to infection and cancer, thus this has an impact for the application of scRNA‐seq in public and prevention health. State‐specific drivers and TCF1^+^ progenitor‐like populations differ between experimental acute and chronic viral infections at an early stage, while dysfunctional states and clusters of CD8 T cells between cancer and infection are shown by transposase‐accessible chromatin sequencing and scRNA‐seq.^13^ This mechanism‐associated information can assist in clinical decision‐making and in designing of new therapeutic strategies for different diseases.

The comprehensive information from scRNA‐seq significantly improves the understanding and knowledge of the genomic, transcriptomic, and epigenomic landscapes of cells within organs. Challenges persist, for example, scRNA‐seq‐based profiles of circulating cells are difficult to associate with diverse cell subpopulations within organ regions, gene signatures, novel cell markers, and functional differences of tissue cells. There are great needs for pre‐clinical and clinical advances to uncover specific brain cell cluster functions of various cell types that are challenging because of the complexity of the organ in development, health, and disease. The transcriptomic profiles of cerebrospinal fluid single cells might be comparable with those of circulating tumor cells, indicating spatial heterogeneity of metastatic sites, cell‐cycle gene, and cancer‐testis antigen profiles, although there are clear heterogeneities among single cells in the same or different origins and locations.^14^ Of liquid biopsies, scRNA‐seq will have more values and impacts if the clusters and transcriptomes of circulating cells could mirror the proportion and nature of cells harvested from “the third cavity”, including the cerebrospinal, thoracic, abdominal, and joint cavities.

Another challenge is that the cell biological behaviors within the microenvironment are dynamic processes of cell‐cell communication and interaction that are difficult to capture by static analytic approaches. Multiple protein factors and cells, pathways, and metabolisms contribute to the formation of the microenvironmental ecosystem and to the maintenance of cell survival, proliferation, and migration.^15^ The combination of scRNA‐seq and spatial transcriptomics is an important approach to define molecular mechanisms of cell‐cell communication in situ and to bridge cell transcriptomic profiles with clinical image phenomes in spatiotemporal molecular images and medicine.^16^, ^17^, ^18^ It is important for the clusters and differentiations of circulating cells to capture the association of infiltrated leukocytes, reflect the landscape of microenvironment inflammatory cells, or more importantly, for circulating single‐cell profiles to represent dynamic functions of tissue inflammatory cells during cell‐cell communication. By integrating scRNA‐seq with single‐cell multi‐omics and trans‐omics and by incorporating artificial intelligence and computational science, clusters, differentiations, and transcriptomic profiles of circulating cells may have the potential to predict the interactions of microenvironmental inflammatory cells, drug sensitivity, and prognosis.^19^, ^20^

scRNA‐seq is on track for use as a routine measurement of clinical biochemistry and to assist in clinical decision‐making and guide the performance of molecular medicine, but there are still a large number of challenges to be overcome. In conclusion, scRNA‐seq‐based clusters and differentiations of circulating blood cells have been examined and informative in patients with various diseases, although the information generated from scRNA‐seq varies between different conditions, technologies, and diseases. Most of the clinical studies published have focused on the landscape of circulating immune cells, disease‐specific patterns of new clusters, understanding of potential mechanisms, and potential correlation between cell clusters, differentiations, cell interactions, and circulating and migrated cells. It is clear that the information from scRNA‐seq advances the understanding of the disease, identifies disease‐specific target panels, and suggests new therapeutic strategies. The adaptation of scRNA‐seq as a routine clinical measurement will require standardization and normalization of scRNA‐seq‐based comprehensive information and validation in a large population of healthy and diseased patients. The integration of public databases on human circulating cell clusters and differentiations with the application of artificial intelligence and computational science will accelerate the application of scRNA‐seq for clinical practice. Thus, we call special attention from scientists and clinicians to the clinical and translational discovery, validation, and medicine opportunities of scRNA‐seq development.
